# High Hepatitis B Surface Antigen Levels Predict Insignificant Fibrosis in Hepatitis B e Antigen Positive Chronic Hepatitis B

**DOI:** 10.1371/journal.pone.0043087

**Published:** 2012-08-20

**Authors:** Wai-Kay Seto, Danny Ka-Ho Wong, James Fung, Philip P. C. Ip, John Chi-Hang Yuen, Ivan Fan-Ngai Hung, Ching-Lung Lai, Man-Fung Yuen

**Affiliations:** 1 Department of Medicine, The University of Hong Kong, Queen Mary Hospital, Hong Kong, Hong Kong; 2 Department of Pathology, The University of Hong Kong, Queen Mary Hospital, Hong Kong, Hong Kong; 3 State Key Laboratory for Liver Research, University of Hong Kong, Queen Mary Hospital, Hong Kong, Hong Kong; Hannover Medical School, Germany

## Abstract

**Introduction:**

There is no data on the relationship between hepatitis B surface antigen (HBsAg) levels and liver fibrosis in hepatitis B e antigen (HBeAg)-positive patients with chronic hepatitis B (CHB).

**Methods:**

Serum HBsAg and HBV DNA levels in HBeAg-positive CHB patients with liver biopsies were analyzed. The upper limit of normal (ULN) of alanine aminotransferase (ALT) was 30 and 19 U/L for men and women respectively. Histologic assessment was based on Ishak fibrosis staging for fibrosis and Knodell histologic activity index (HAI) for necroinflammation.

**Results:**

140 patients (65% male, median age 32.7 years) were recruited. 56 (40%) had ALT ≤2×ULN. 72 (51.4%) and 42 (30%) had fibrosis score ≤1 and necroinflammation grading ≤4 respectively. Patients with fibrosis score ≤1, when compared to patients with fibrosis score >1, had significantly higher median HBsAg levels (50,320 and 7,820 IU/mL respectively, p<0.001). Among patients with ALT ≤2×ULN, serum HBsAg levels achieved an area under receiver operating characteristic curve of 0.869 in predicting fibrosis score ≤1. HBsAg levels did not accurately predict necroinflammation score. HBsAg ≥25,000 IU/mL was independently associated with fibrosis score ≤1 (p = 0.025, odds ratio 9.042).Using this cut-off HBsAg level in patients with ALT ≤2×ULN, positive and negative predictive values for predicting fibrosis score ≤1 were 92.7% and 60.0% respectively. HBV DNA levels had no association with liver histology.

**Conclusion:**

Among HBeAg-positive patients with ALT ≤2×ULN, high serum HBsAg levels can accurately predict fibrosis score ≤1, and could potentially influence decisions concerning treatment commencement and reduce the need for liver biopsy.

## Introduction

Chronic hepatitis B (CHB) is known for its highly variable disease course, ranging from an inactive carrier state to the development of clinical complications, including cirrhosis and hepatocellular carcinoma (HCC) [1]. CHB patients with repeated hepatitis flares were noted to have increased necroinflammation in liver histology, leading to increased fibrogenesis and subsequent disease progression [2].

Treatment guidelines by two international liver associations [3], [4] recommend treatment commencement when serum alanine aminotransferase (ALT) is persistently >2×upper limit of normal (ULN) in hepatitis B e antigen (HBeAg)-positive patients. Guidelines from another international liver association recommend treatment when there is clinical evidence of significant liver fibrosis e.g. by using liver biopsy in patients with elevated ALT [5]. There is increasing evidence that patients with ALT ≤2×ULN could still eventually develop clinical complications [6]. HBeAg-positive patients with normal ALT are traditionally classified as in the immune tolerant phase of disease, with minimal histologic changes on liver biopsy [7]. However histologic studies of HBeAg-positive patients with “high normal” ALT have been shown to have significant fibrosis and necroinflammation [8], [9]. In addition, the definition of “normal ALT” has also been re-evaluated. One study of 6835 healthy blood donors has suggested lowering the ULN of ALT to 30 U/L for men and 19 U/L for women [10]. A study from Asia involving 1105 potential liver donors also has similar recommendations [11]. Because of all these controversial issues, using ALT levels to classify patients for treatment initiation is suboptimal. Assessment of fibrosis is thus an important parameter in deciding treatment.

Given the invasive nature of liver biopsy, several non-invasive indices have been developed for the prediction of significant fibrosis in CHB [12], [13], [14]. These studies however are limited by their small sample sizes, the lack of large-scale external validation, and the use of serum markers not routinely available from standard laboratories. The use of predictive models established in chronic hepatitis C has also produced conflicting results [15], [16]. Transient elastography is another method for assessing liver fibrosis [17]. However, it is often difficult to determine in obese patients, has reduced diagnostic accuracy with lower fibrosis scores, and is affected by even small degrees of ALT elevation, with 40–50% of patients still requiring other means of fibrosis assessment [17].

The quantification of hepatitis B surface antigen (HBsAg) levels has been recently advocated as a surrogate marker for intrahepatic closed covalently circular DNA (cccDNA) [18]. Recent evidence have shown serum HBsAg levels to be useful in identifying inactive CHB carriers [19], predicting subsequent HBsAg seroclearance [20], [21], [22], and predicting favorable outcomes with pegylated interferon therapy [23]. Serum HBsAg levels have been shown to be extremely high among HBeAg-positive patients with normal ALT [24], [25], and it has been suggested high HBsAg levels could be supportive evidence of immune tolerance [26]. The aim of our study was to evaluate the use of serum HBsAg levels in assessing liver histology in HBeAg-positive CHB patients.

## Methods

### Ethics Statement

The present study was approved by the Institutional Review Board, the University of Hong Kong and West Cluster of Hospital Authority, Hong Kong. All patients had written consent prior to liver biopsy and study entry with all clinical investigation conducted according to the principles expressed by the Declaration of Helsinki.

### Patients

The present study included treatment-naive HBeAg-positive CHB patients who were recruited for therapeutic drug trials between 1994 to 2008 in the Department of Medicine, the University of Hong Kong, Queen Mary Hospital. All patients were HBsAg-positive for at least 6 months before study entry. Other inclusion criteria included ALT <10×ULN and HBV DNA ≥20,000 IU/mL. Patients with concomitant liver diseases, including chronic hepatitis C or D infection, Wilson’s disease, autoimmune hepatitis, primary biliary cirrhosis, significant intake of alcohol (30 grams per day for male, 20 grams per day for female) and decompensated liver disease were excluded.

### Liver Biopsy

Two different biopsy needles were used. An 18G sheathed cutting needle (Temno Evolution, Cardinal Health, McGaw Park, IL) was used in 58 patients, while a 17G core aspiration needle (Hepafix, B. Braun Melsungen AG, Germany) was used for the remaining 82 patients. The biopsy lengths were 1.5 to 1.8 cm and 2 to 5 cm respectively. A single pathologist (initials PPCI), blinded to all biochemical, serologic and virologic parameters, was assigned to review all biopsy specimens. Biopsies were fixed, paraffin-embedded, and stained with hematoxylin and eosin for morphological evaluation and Masson’s trichrome stain for assessment of fibrosis. Histologic staging of fibrosis and grading of necroinflammation was performed using the Ishak fibrosis score (range 0 to 6) [27] and Knodell histologic activity index (HAI) (range 0 to 18) [28] respectively. “Insignificant fibrosis” was defined as an Ishak fibrosis score of equal or less than 1. “Insignificant necroinflammation” was defined as a Knodell HAI score of equal or less than 4.

### Laboratory Assays

Serum samples used for measurements were taken at the day of biopsy and stored at −20°C. Following recommendations of current treatment guidelines [3], the ULN of serum ALT was defined as 30 U/L for men and 19 U/L for women [10]. Serum HBsAg, HBeAg and antibody to the hepatitis B e antigen (anti-HBe) were measured using commercially available immunoassays (Abbott Laboratories, Chicago, IL). Serum HBV DNA levels were measured using Cobas Taqman assay (Roche Diagnostics, Branchburg, NJ), with a linear range of 20 to 1.98×10^8^ IU/mL. Samples with HBV DNA levels higher than 1.98×10^8^ IU/mL were diluted at 1∶100 for retesting. Serum HBsAg titer was measured using the Elecsys HBsAg II assay (Roche Diagnostics, Gmbh, Mannheim), with a linear range of 0.05 to 52,000 IU/mL. Samples with HBsAg levels higher than 52,000 IU/mL were retested at a dilution of 1∶100.

### Statistical Analysis

All continuous variables are expressed in median (range). Statistical analyses were performed using SPSS version 18.0 (SPSS Inc, Chicago, Illinois). The Mann-Whitney U test was used for comparing continuous variables with a skewed distribution; Chi squared test was used for categorical variables. Correlation was performed using Spearman’s bivariate correlation. The predictions of minimal histologic changes were first examined by the construction of corresponding receiver operating characteristic (ROC) curves, followed by the assessment of overall accuracy by areas under the curves (AUCs). The Youden Index, defined as the sensitivity plus the specificity minus one, was used to identify the optimal level of prediction. Multivariate logistic regression was used to identify factors independently associated with insignificant fibrosis. A two-sided p value of <0.05 was considered statistically significant.

## Results

One hundred and forty HBeAg-positive patients were included in the present study. The baseline demographics are shown in Table 1. Three patients (2.1%) had histologic evidence of cirrhosis. Based on liver biochemistry, 17 (12.1%) were classified as immune tolerant with normal ALT; the remaining 123 (87.9%) were classified to be in immune clearance. There were no significant differences in age, gender, liver biochemistry and serum HBV DNA between the two groups of patients (Table 1, all p>0.05).

**Table 1:** Baseline characteristics of all 140 patients.

	All patients (n = 140)	ALT ≤2×ULN (n = 56)	ALT >2×ULN (n = 84)	p value*
Age	32.7 (16.6–60.1)	32.6 (16.6–55.0)	32.5 (18.0–60.1)	0.309
Number of male patients	91 (65.0%)	40 (71.4%)	51 (60.7%)	0.193
Albumin (U/L)	46 (37–54)	47 (39–52)	45 (37–54)	0.152
Bilirubin (umol/L)	10 (3–31)	9.5 (4–31)	10.5 (3–30)	0.088
ALT (U/L)	67.5 (14–175)	38 (14–60)	89 (46–175)	<0.001
HBV DNA (log IU/mL)	7.96 (4.41–12.4)	8.14 (4.83–11.9)	7.72 (4.41–12.4)	0.113
HBsAg (IU/mL)	17,680 (62–319,800)	52,535 (257–319,800)	9,362 (62–217,200)	<0.001

All continuous values expressed in median (range).

ALT, alanine aminotransferase; ULN, upper limit of normal; HBsAg, hepatitis B surface antigen.

ALT upper limit of normal: 30 U/L for men, 19 U/L for women.

* Comparison was between patients with ALT ≤2×ULN and ALT >2×ULN.

**Figure 1:**
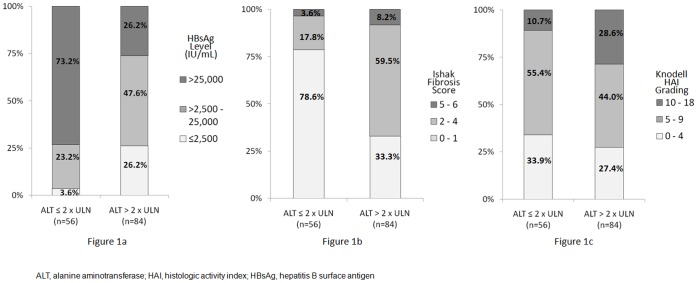
Distribution of all patients stratified by ALT levels. HBsAg levels (Figure 1a), fibrosis scores (Figure 1b) and necroinflammation gradings (Figure 1c) of all patients are shown.

Median HBsAg levels for patients with normal ALT, ALT 1–2×ULN and ALT >2×ULN were 105,020 IU/mL (range: 13,490–319,800 IU/mL), 40,490 IU/mL (range: 257–286,300 IU/mL) and 9,362 IU/mL (range: 62–217,200 IU/mL) respectively (p<0.001). The distribution of HBsAg levels among patients stratified by ALT ≤2×ULN versus ALT >2×ULN is shown in Figure 1a. A significantly larger proportion of patients with ALT ≤2×ULN had serum HBsAg >25,000 IU/mL when compared to patients with ALT >2×ULN (73.2% versus 26.2%, p<0.001).

Serum HBsAg showed moderate correlation with serum HBV DNA levels (r = 0.403, p<0.001), and moderate inverse correlation with ALT levels (r = −0.450, p<0.001).

### Liver Histology

**Figure 2:**
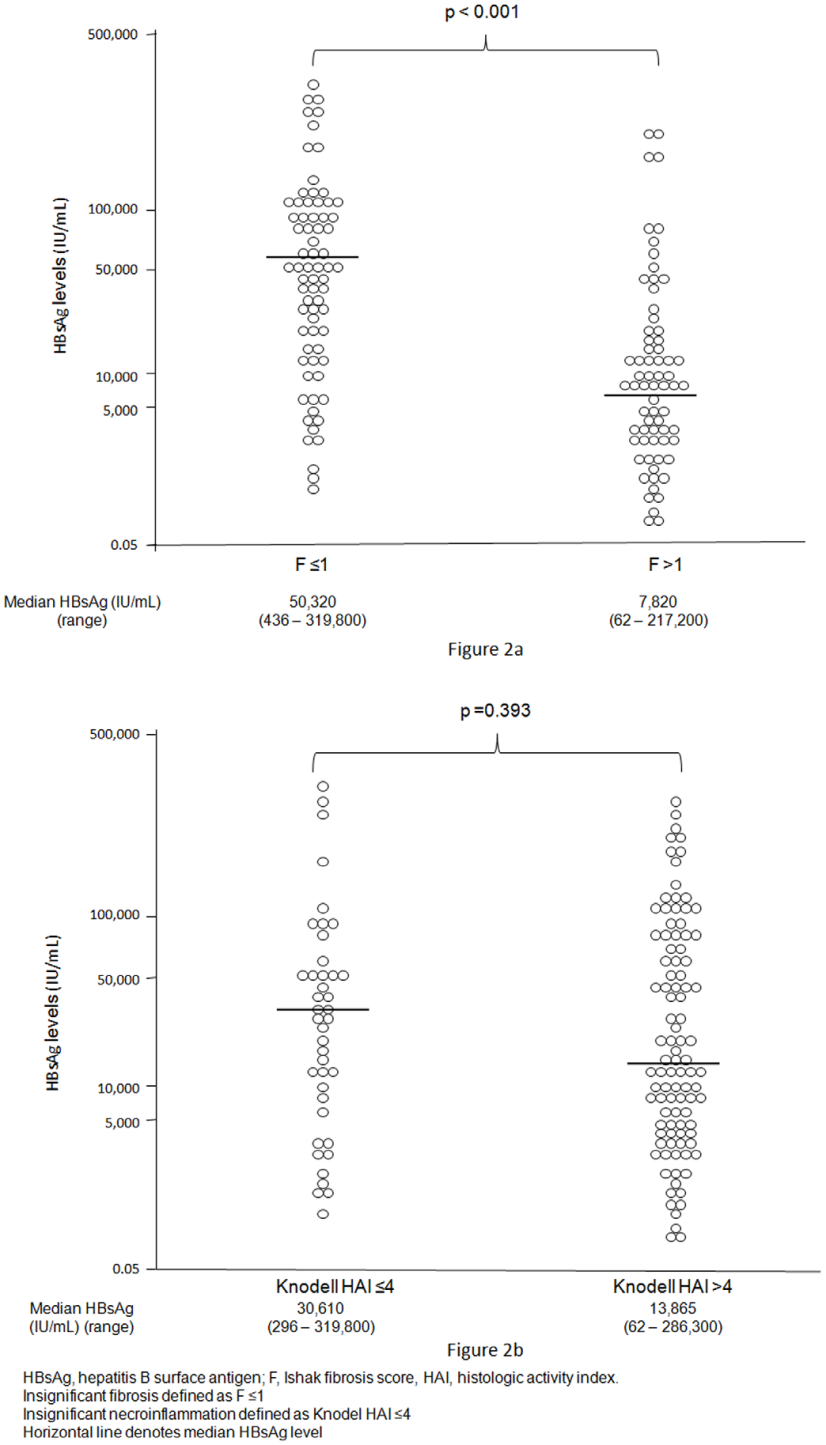
Distribution of serum HBsAg levels. Patients are stratified by fibrosis scores (Figure 2a) and necroinflammation gradings (Figure 2b).

The distribution of fibrosis scores and necroinflammation gradings of all 140 patients stratified by ALT levels is shown in Figures 1b and 1c. Seventy-two (51.4%) and 42 (30%) patients had insignificant fibrosis and necroinflammation respectively. All immune tolerant patients with normal ALT (n = 17) had insignificant fibrosis. Among patients with ALT ≤2×ULN (n = 56), 44 (78.6%) had insignificant fibrosis, significantly more than among patients with ALT >2×ULN (33.3%, p<0.001). The proportion of patients with insignificant necroinflammation among the two patients groups was similar (33.9% and 27.4% respectively, p = 0.408). The type of biopsy needle used (i.e. the sheathed cutting needle versus the core aspiration needle) did not influence the degree of fibrosis and necroinflammation (p = 0.735 and 0.970 respectively). Serum HBsAg levels among all patients divided by their histologic scores and gradings are shown in Figures 2a and 2b. Patients with insignificant fibrosis had significantly higher median HBsAg levels (p<0.001). In the subgroup of 39 patients with ALT 1–2×ULN, median HBsAg levels were also significantly higher in those with insignificant fibrosis (51,400 IU/mL, range: 2,598 to 286,300 IU/mL) when compared to those with significant fibrosis (7,703 IU/mL, range 257 to 78,810 IU/mL) (p = 0.002). Comparing patients with insignificant necroinflammation and those with significant necroinflammation, median HBsAg levels showed no significant difference (p = 0.393).

Median serum HBV DNA levels showed no significant difference in patients with insignificant fibrosis compared to patients with significant fibrosis for the whole group (8.01 and 7.85 log IU/mL respectively, p = 0.794) and for the subgroup of patients with ALT ≤2×ULN (8.23 and 7.65 log IU/mL respectively, p = 0.318). There was also no significant difference in median HBV DNA levels among patients with insignificant necroinflammation versus significant necroinflammation for the whole group of patients (7.91 and 7.99 log IU/mL respectively, p = 0.897) and for the subgroup of patients with ALT ≤2×ULN (8.40 and 8.02 log IU/mL respectively, p = 0.095).

Serum HBsAg showed a moderate inverse correlation with fibrosis scores (r = −0.449, p<0.001). Serum HBsAg also had an inverse correlation with necroinflammation gradings, but with a lower correlation coefficient (r = −0.269, p = 0.001). Serum HBV DNA had no correlation with both fibrosis scores (r = −0.076, p = 0.373) and necroinflammation gradings (r = −0.042, p = 0.624).

### Predictive Value of HBsAg for Minimal Histologic Changes

**Figure 3:**
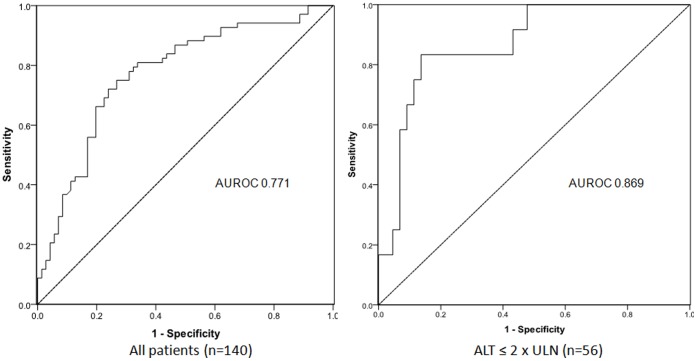
Receiver operating characteristic curves of serum HBsAg levels in predicting insignificant fibrosis (Ishak fibrosis score ≤1).

The ROC curves and the AUC values of serum HBsAg levels in predicting insignificant fibrosis and necroinflammation are depicted in Figure 3 and Table 2. Serum HBsAg levels produced a better AUC for insignificant fibrosis in patients with ALT ≤2×ULN (AUC 0.869) compared to the overall population (AUC 0.771). Serum HBsAg levels did not have any predictive value for insignificant necroinflammation (AUC 0.546 and 0.532 for all patients and patients with ALT ≤2×ULN respectively).

**Table 2:** Area under the receiver operating characteristic curve of serum HBsAg levels in predicting minor histologic changes.

		AUC	Standard Error	p value	95% CI
Insignificant fibrosis (F ≤1)	All patients (n = 140)	0.771	0.040	<0.001	0.692–0.851
	ALT ≤2×ULN (n = 56)	**0.869**	0.054	<0.001	0.763–0.976
Insignificant necroinflammation (Knodell HAI ≤4)	All patients (n = 140)	0.546	0.053	0.393	0.442–0.650
	ALT ≤×2 ULN (n = 56)	0.532	0.080	0.697	0.376–0.698

F, Ishak fibrosis score; HAI, histologic activity index; ALT, alanine aminotransferase; ULN, upper limit of normal; AUC, area under curve; CI, confidence interval.

The sensitivity, specificity and predictive values of different HBsAg levels in predicting insignificant fibrosis among patients with ALT ≤2×ULN are shown in Table 3. Based on the Youden Index, the optimal level of serum HBsAg to predict insignificant fibrosis was ≥27,490 IU/mL (Youden index 0.697, sensitivity 86.4%, specificity 83.3%). Rounding off to the nearest five-thousandth level, serum HBsAg ≥25,000 IU/mL was able to predict insignificant fibrosis with a sensitivity of 86.4%, specificity of 75.0%, positive predictive value of 92.7% and negative predictive value of 60.0%. The 7.3% (3 out of 41) of patients with HBsAg ≥25,000 IU/mL but fibrosis score >1 had only stage 2 fibrosis. Serum HBsAg ≥100,000 IU/mL was 100% predictive of insignificant fibrosis.

**Table 3:** Sensitivity, specificity and predictive values of different HBsAg levels for predicting insignificant fibrosis among patients with ALT ≤2×ULN (n = 56).

HBsAg (IU/mL)	Number of patients	Sensitivity	Specificity	*PPV*	NPV	LR+	LR−
≥10,000	45	90.9%	58.3%	***88.9%***	63.6%	2.18	0.16
≥25,000	41	86.4%	75.0%	***92.7%***	60.0%	3.46	0.18
≥50,000	32	68.2%	83.3%	***93.8%***	41.7%	4.08	0.38
≥75,000	24	52.3%	91.7%	***95.8%***	34.4%	6.30	0.52
≥100,000	16	36.4%	100%	***100%***	30.0%	–	0.64

Insignificant fibrosis defined as Ishak fibrosis score ≤1.

HBsAg, hepatitis B surface antigen; ALT, alanine aminotransferase; ULN, upper limit of normal; PPV, positive predictive value; NPV, negative predictive value, LR+, positive likelihood ratio; LR-, negative likelihood ratio.

### Multivariate Analysis for Predicting Insignificant Fibrosis

Among patients with ALT ≤2×ULN, younger age (p<0.001), serum HBsAg ≥25,000 IU/mL (p<0.001) and lower serum ALT (p = 0.001) were associated with insignificant fibrosis by univariate analysis. The multivariate analysis of factors independently predictive of insignificant fibrosis is shown in Table 4. After adjusting for different clinical parameters, factors independently associated with insignificant fibrosis included serum HBsAg ≥25,000 IU/mL (p = 0.025, odds ratio 9.042, 95% confidence interval 1.325–61.716) and younger age (p = 0.030).

**Table 4:** Multivariate analysis of factors independently associated with insignificant fibrosis among patients with ALT ≤2×ULN.

	p value	Odds ratio	95% Confidence Interval
***HBsAg ≥25,000 IU/mL***	***0.025***	***9.042***	1.325–61.716
Age (Years)	0.030	0.884	0.791–0.988
ALT (U/L)	0.078	0.936	0.869–1.007

Insignificant fibrosis defined as Ishak fibrosis score ≤1.

ALT, alanine aminotransferase; HBsAg, hepatitis B surface antigen.

## Discussion

An elevated ALT level classically differentiates immune clearance from immune tolerance in HBeAg-positive CHB. Nevertheless, studies have shown ALT to be an inaccurate marker of liver injury [7], [9]. Although HBsAg staining patterns in liver histology [29] and antibody to the hepatitis B core antigen IgM titers [30] could assist in differentiating the two HBeAg-positive disease phases, the assessment of fibrosis remains an essential step in deciding treatment commencement [5]. Current non-invasive methods are unable to accurately identify patients with severe histologic abnormalities. Our present study showed serum HBsAg levels can play an important role in identifying HBeAg-positive patients with insignificant fibrosis and potentially reduce the need for liver biopsies. Although there had been preliminary analysis linking HBsAg levels with histologic severity [31], our study to our knowledge was the first to formally use liver histology as an outcome measure to assess the role of HBsAg titers in distinguishing insignificant and significant fibrosis.

The present study identified two serum HBsAg cut-off levels useful for predicting insignificant fibrosis among HBeAg-positive patients with ALT ≤2×ULN. Serum HBsAg ≥100,000 IU/mL was 100% predictive of insignificant fibrosis. Prior studies also found similarly high serum HBsAg levels in immune tolerant patients defined by normal ALT levels [24], [25], [32]. HBeAg-positive patients with HBsAg ≥100,000 are likely to have insignificant fibrosis even if ALT levels are minimally elevated.

The present study also found the optimal serum HBsAg cut-off level to predict insignificant fibrosis to be of ≥25,000 IU/mL. Among HBeAg-positive patients with ALT ≤2×ULN, serum HBsAg ≥25,000 IU/mL had a positive predictive value of 92.7% of predicting insignificant fibrosis. In addition, serum HBsAg ≥25,000 IU/mL was the best factor independently associated with insignificant fibrosis (p = 0.025, odds ratios 9.042). Our results suggest that HBeAg-positive patients with ALT ≤2×ULN and serum HBsAg ≥25,000 IU/mL can be observed without the need of liver biopsies. If serum HBsAg levels are below 25,000 IU/mL, other forms of assessment of fibrosis are necessary to decide for the commencement of therapy.

Our study failed to establish any association between serum HBV DNA levels and liver histology in HBeAg-positive patients. A possible explanation is that in these patients, the immune-mediated response during immune clearance may lead to fluctuating viremic levels with varying degrees of abnormalities in histology [33]. Several non-invasive predictive indices involving HBeAg-positive patients proposed in recent studies have also not included serum HBV DNA as a factor for prediction [13], [14]. Serum HBV DNA levels are of greater predictive value in HBeAg-negative patients [34].

While the exact mechanism for the inverse relationship between the HBsAg levels and the degree of fibrosis remains to be examined, it may be related to the different stages of immune clearance. HBsAg is found extensively in immunohistochemical staining of liver histology in the immune tolerance phase [29]. With the transition from immune tolerance to early immune clearance, the immune system starts to increase its magnitude of immune control on the HBV. Serum HBsAg levels still remain high at the transition from immune tolerance to early immune clearance phase (ALT level may be at the high normal range) and it is to be expected that there will be minimal fibrosis because immune mediated attack is still of low magnitude. Upon entering into a more full-blown stage of immune clearance with repeatedly greater immune mediated damage, more fibrosis develops, and viral control is achieved with decreasing HBsAg levels. HBsAg production could also be influenced by the development of preS/S mutants during immune clearance [35].

Intriguingly, high HBsAg levels are not always favorable in CHB, as shown by recent studies demonstrating high HBsAg levels to be associated with the development of HCC [36], [37]. Hence, further longitudinal studies should be performed to examine the exact relationship between severity of fibrosis, disease progression and HBsAg levels by serial HBsAg measurement. Studies including non-Asian CHB patients would also be important, since such patients are infected later in life, with the classical immune tolerant phase absent or very short, and could well demonstrate different results.

Our study employed the lowered ULN for ALT (30 U/L for men, 19 U/L for women) as recommended by current treatment guidelines. Therefore, our cohort of patients with ALT ≤2×ULN accurately represents the HBeAg-positive population in which assessment of histologic severity is essential before deciding on treatment. In addition, the assay used for serum HBsAg measurement in our study has a broad dynamic range, minimizing the potential errors related to manual dilution in the measurement of high levels. Our study is limited by the lack of non-Asian CHB patients. In addition, HBV genotyping was not performed in our study, although prior studies have shown genotypes B and C, the two common genotypes in Hong Kong, have a similar risk of advanced fibrosis and histologic progression [38], [39]. Future studies involving different CHB populations with different genotypes are required to validate our findings. The comparison of the predictive value of HBsAg levels (including for HBeAg-negative histology) with different non-invasive predictive indices (e.g. the asparate aminotransferase/platelet ratio index) and transient elastography are also needed. In additional, future clinical and cost-effectgive studies with larger cohorts, after the adjustment of HBV genotype, could consider fitting HBsAg levels and other available non-invasive markers as an algorithm for practical clinical usage.

In conclusion, serum HBsAg ≥25,000 IU/mL was independently associated with insignificant fibrosis. This level accurately predicted insignificant fibrosis in HBeAg-positive CHB patients with ALT ≤2×ULN (AUROC 0.869, positive predictive value 92.7%), the group of patients in which histologic evaluation is recommended. Measurement of serum HBsAg levels can thus assist treatment decisions among HBeAg-positive patients and potentially reduce the need for liver biopsies.
